# Effect of avocado/soybean unsaponifiables on periodontal repair in rats with arthritis and induced periodontitis

**DOI:** 10.1590/1678-7757-2018-0602

**Published:** 2019-08-30

**Authors:** Jackeline do Nascimento Tsurumaki, Luiz Guilherme Freitas de Paula, Sabrina Garcia de Aquino, Elcio Marcantonio, Guilherme José Pimentel Lopes de Oliveira, Rosemary Adriana Chiérici Marcantonio

**Affiliations:** 1 Universidade Estadual Paulista Universidade Estadual Paulista Faculdade de Odontologia de Araraquara Departamento de Diagnóstico e Cirurgia Araraquara São Paulo Brasil Universidade Estadual Paulista (UNESP), Faculdade de Odontologia de Araraquara, Departamento de Diagnóstico e Cirurgia, Araraquara, São Paulo, Brasil; 2 Centro Universitário de Anápolis Centro Universitário de Anápolis Faculdade de Odontologia Anápolis Goiás Brasil Centro Universitário de Anápolis, Faculdade de Odontologia (Unievangélica), Anápolis, Goiás, Brasil; 3 Universidade Federal da Paraíba Universidade Federal da Paraíba Faculdade de Odontologia Departamento de Ciências da Saúde João Pessoa Paraíba Brasil Universidade Federal da Paraíba, Faculdade de Odontologia, Departamento de Ciências da Saúde, João Pessoa, Paraíba, Brasil; 4 Universidade Federal de Uberlândia Universidade Federal de Uberlândia Faculdade de Odontologia Departamento de Periodontia Uberlândia Minas Gerais Brasil Universidade Federal de Uberlândia, Faculdade de Odontologia, Departamento de Periodontia, Uberlândia, Minas Gerais, Brasil

**Keywords:** Arthritis, Bone regeneration, Drug therapy, Periodontitis

## Abstract

**Objective:**

This study aimed to evaluate the effect of avocado/soybean unsaponifiables (ASU) on periodontal repair in rats with induced periodontitis and arthritis.

**Methodology:**

Forty-five rats were submitted to periodontitis induction by insertion of ligatures into the upper second molars, maintained for 15 days. These animals were randomly allocated to 3 groups according to the presence of induced arthritis (ART) and the application of the ASU: Control (CTR) group-healthy animals, where saline solution was administered; ART-animals with induced arthritis, where saline solution was administered; ART/ASU-animals with induced arthritis, where ASU (0.6 mg/ kg) was administered. The drugs were administered daily by gavage and the animals were euthanized after 7, 15 and 30 days of the ligature removal. Bone resorption, inflammatory infiltrate composition and marker proteins expression of the differentiation and formation of osteoclasts (RANKL and TRAP) were assessed.

**Results:**

The ART/ASU group presented higher bone volume than the ART group at 7 and 30 days after the ligature removal. Furthermore, the ART group presented higher quantity of inflammatory cells and expression of TRAP and RANKL than the other groups.

**Conclusion:**

ASU administration improves the repair of periodontal tissues in an experimental periodontitis model in rats with induced arthritis.

## Introduction

The supporting periodontium is the tissue responsible for the insertion of the tooth in the maxillary bones, and this tissue is composed of cementum, periodontal ligament and the alveolar bone.^1^ The destruction of the supporting periodontium occurs during the progression of periodontal disease (PD) characterized by an inflammatory response against the microbial challenge that induces the expression of proinflammatory cytokines, which stimulate osteoclastogenesis and consequently the bone resorption.^1^^,^^2^ The PD, rheumatoid arthritis (ART) and osteoarthritis (OA) are immuno-inflammatory diseases characterized by inflammatory process-induced bone resorption.^3^^,^^4^ Although these diseases have different etiological factors, all share risk factors and similar pathogenic mechanisms.^1^^,^^3^^,^^4^ Thus, drugs used for the treatment of ART and OA have been indicated as host response modulating agents, which can be used as adjuncts in periodontal therapy.^1^

Avocado/soybean unsaponifiables (ASU) have been indicated for the treatment of ART and OA and have been shown to reduce the inflammatory symptoms caused by these diseases. Clinical and pre-clinical studies have demonstrated that this drug can modify the structural patterns of the synovial joints presenting bone resorptions.^5^^–^^9^ This action on the proliferation of connective tissue associated with the reduction of biological pro-inflammatory mediators expression has raised interest in the use of ASU in periodontal treatment.^10^^,^^11^ Pre-clinical studies showed that ASU improved the periodontal repair and treatment outcomes of induced periodontitis,^10^^,^^11^ as well as improving the osseointegration patterns of implants; ^12^these effects were related with the reduction of pro-inflammatory biological mediators expression, such as Interleukin 1 beta (IL-1β), Receptor activator of nuclear factor kappa-B ligand (RANKL), and Tartrateresistant acid phosphatase (TRAP),^10^ and enhanced in the expression of growth factors, such as Bone morphogenetic protein 2 (BMP2) and Transforming growth factor beta 1 (TGFβ1).^12^

It has been proposed that the PD treatment with host response modulators has a more expressive effect in patients who present some systemic condition that changes the progression of tissue destruction or the response to periodontal therapy.^2^^,^^13^ For example, compared to healthy individuals, patients with ART have more severe PD, with higher levels of periodontal inflammation, more dental loss, higher frequency of sites with advanced insertion loss and deeper periodontal pockets;^14^^,^^15^ thus, patients with ART and PD can benefit from anti-arthritic therapy with ASU, because this drug has a mechanism for blocking inflammatory processes associated with the stimulation of connective tissue proliferation.^5^^,^^6^ Due to this effect, this study aims to evaluate the effect of ASU administration on periodontal repair in animals with induced arthritis and periodontitis.

## Methodology

This study was submitted and approved by the Animal Use Ethics Committee of our institution (09/2012). In this study, 45 male rats (*Rattus norvegicus*), albinus variation, Holtzman, weighing 300-330 g were used. The animals were kept in a room with controlled light and temperature, fed with solid feed and had water access *ad libitum*, before, during and throughout the experimental period. The animals were maintained in polypropylene cages with 4 animals in each cage. The entire study was conducted according to ARRIVE guidelines.

### Groups

The animals were randomly allocated in 3 groups according to systemic condition and the ASU use: Group CTR – Healthy animals submitted to saline solution administration; Group ART – Animals with induced arthritis submitted to the saline solution administration; and Group ART/ASU – Animals with induced arthritis who underwent ASU administration (0.6 mg/kg).^10^ The animals received ASU or saline solution from the day of induction of periodontal disease. This administration occurred daily through gavage until the day of the animals’ euthanasia, which occurred 7, 15 and 30 days after the ligature removal (n=5 animals *per* group/period).

### Induction of experimental arthritis

**Figure 1:**
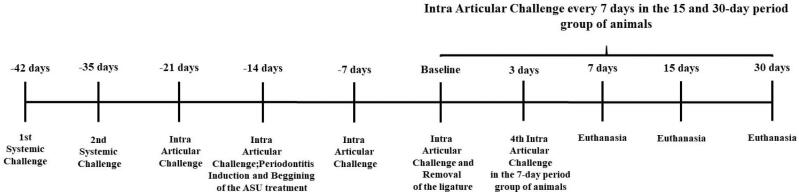
Flowchart of the experiment. Two systemic challenges were achieved with mBSA within 7 days. Fourteen days after the last systemic challenge, the application of the antibody against mBSA within the knee joints was started every 7 days. On the day of the second intra-articular challenge, the induction of periodontal disease was performed by ligatures that were held in place for 15 days. After removing the ligatures, the animals were submitted to euthanasia at 7, 15 and 30 days

The experimental-induced arthritis model was conducted by administration of antigens and was performed in 2 challenges:^16^ 1) Subcutaneous challenge and 2) intra-articular challenge. The subcutaneous challenge was achieved through the administration of methylated bovine serum albumin (mBSA). The animals were sensitized with 2 subcutaneous applications of 500 μg mBSA (Sigma, St. Louis, Missouri, USA), diluted in 0.2 mL of emulsion containing 0.1 mL of saline and 0.1 mL of complete Freund‘s adjuvant solution (Sigma, St. Louis, Missouri, USA), with a 7-day interval between applications. Nonimmunized animals received saline injections in the same periods as the immunized animals. Intraarticular challenge was performed 14 days after the last mBSA subcutaneous application, and arthritis was induced by intra-articular application of the mBSA antigen (10 μg/joint cavity), diluted in 10 μL of PBS in the right posterior paw joint. The applications were performed at 7-day intervals until the animals were euthanized. In the animals that were euthanized at the 7-day period, the fourth application of the intraarticular challenge was performed 3 days after the removal of the ligatures. Animals that did not receive the intra-articular challenge received injections with saline solution (Figure 1).

### Induction of the PD

The animals were anesthetized by a combination of ketamine and xylazine at a ratio of 0.08 mL/100 g body weight of ketamine (Virbac do Brasil Ind. Com. Ltda, São Paulo, S P, Brazil) and 0.04 ml/100 g body weight of xylazine (Virbaxyl 2%, Virbac do Brasil Ind. E Com. Ltda, São Paulo, S P, Brazil). Subsequently, the ligatures (cotton yarn) were inserted in the sub gingival region around the both upper second molars. After a period of 15 days, the bandages were removed and the administration of the drugs began (Figure 1).

### Sample retrieval

After 7, 15 and 30 days subsequent to ligature removal and initiation of the treatment with the drugs, the animals were submitted to euthanasia by anesthetic overdose. The jaws and right posterior paw joint of each animal were removed and fixed in 4% paraformaldehyde for a 24-hour period.

### Analysis of joints

The joints were evaluated histologically only to confirm the effectiveness of the arthritis induction model. After decalcification in 7% EDTA solution (pH 7.2 in PBS) for 3 months, the samples were processed for paraffin inclusion and execution of the histological sections. Three slides with 3 histological sections were made for each joint, with 6 μm serial coronal sections stained with hematoxylin & eosin (HE). The conditions of synovial membrane thickness, inflammatory infiltrate and presence of cartilage/bone erosion were described.

### Micro CT analysis

After fixation, the maxillas were scanned in a micro CT scan (Skyscan, Aatselaar, Antwerp, Belgium) with the following parameters: Camera pixel: 12.45; power of the X-ray tube: 65 kVP; X-ray intensity: 385 μA; integration time: 300 ms; filter: Al-1 mm; and voxel size: 18 μm. The images were reconstructed, spatially reoriented and analyzed by specific software (NRcom, Data Viewer and CTan, Skyscan, Aatselaar, Antwerp, Belgium). Linear measurements were performed from the cementum-enamel junction to the top of the bone crest at 6 points around the second maxillary molar (two buccal points associated with the medial portion of the mesial and distal roots, two palatine points also associated with the medial portion of the mesial and distal roots, and one point in the medial part of each proximal surface), and the linear measures result of each sample was the mean of these 6 points measurements. Volumetric analyses were also carried out to determine the amount of mineralized tissue (BV/ TV) in the interproximal regions, that started from the cementum-enamel junction of the second molar and the adjacent teeth to the top of the dental apices, and in the furcation region of the second molar, starting from the furcation roof to the top of the dental apices (60 sections). The threshold used in the analysis was 65-255 shades in grayscale, and the BV/TV values were expressed as a percentage. A trained and blinded examiner for the experimental groups performed this analysis (JNT).

### Stereometry

After decalcification of the maxilla samples in 7% EDTA for 8 weeks, the samples were included in paraffin, cut to a thickness of 5 μm and stained by hematoxylin-eosin (HE) technique. Using a DIASTAR optical microscope (Leica Microsystems DFC-300-FX; Leica Reichert & Jung Products, Wetzlar, Hessen, Germany), the histological images were analyzed following 3 sections *per* tooth/animal, which were 60 μm apart.

After capturing the images, a grid with 32400 μm^2^ of area, with 4 squares in height and 9 in length, containing 50 intersection points, was positioned in the proximal regions below the junctional epithelium and the region just below the furcal roof using Adobe Photoshop CS4 software. The stereometric point-counting technique was used in the stained sections to evaluate the proportion of tissue components coincident with the intersection points, in a two-dimensional plane. In this way, the proportion of the following tissue components was determined: extracellular matrix, fibroblasts, inflammatory cells and blood vessels. Then, a percentage of each tissue component was performed in relation to the total number of counted points. This analysis was performed by a trained and blinded examiner for the experimental groups (JNT).

### Immunohistochemistry analysis

The histological sections were mounted on silanized slides, followed by a routine laboratory procedure for deparaffinization and rehydration. Then, the endogenous peroxidase was inactivation performed by applying 3% hydrogen peroxide for 30 minutes, and then blocking the non-specific epitopes was performed by applying the bovine albumin protein (BSA) at 3% for 120 minutes. Then, the sections were incubated for 16 hours on the primary antibodies to tartrate resistant acid phosphatase (TRAP) (1:200) and nuclear activator receptor ĸB/ligand (RANKL) (1:100) (Santa Cruz Biotechnology, Dallas, Texas, USA). As a negative control, histological sections were treated with 1% PBS. Subsequently, the cuts were treated by the avidin-biotin-peroxidase complex and stained by diaminobenzidine. The sections were counterstained with Carrazi hematoxylin solution for cell nuclei visualization. The images were obtained through a camera coupled to a light microscope (Leica Microsystems DFC-300-FX; Leica Reichert & Jung Products, Wetzlar, Hessen, Germany). The TRAP expression was analyzed by cell counts, whereas RANKL expression was analyzed by a marker cell extension score:^10^ (0) No labeling (0% cells); (1) weak (0-25% of cells); (2) moderate (25-50% of cells); (3) strong (>50% of cells). The analysis of the TRAP and RANKL expressions were performed in the interproximal and furcation regions in their full extent by a trained examiner, blinded to the experimental groups (GJO).

### Statistics

A statistical software (Graphpad Prism 6 software, San Diego, California, USA) was used to perform the statistical analysis. The average data of both upper second molars were used in all the analysis as a result of each animal. The data generated by the micro CT, stereometric and TRAP expression analyses were submitted to distribution analysis in relation to the mean by the Kolgomorov-Smirnov normality test. As the data were normally distributed (p<0.05), parametric tests were applied for the inferential data analysis. The one-way ANOVA test, complemented by the Tukey test, was applied to evaluate the data between the groups in each experimental period and within each group by varying the evaluation periods. For the analysis of the RANKL expression, non-parametric Kruskall-Wallis tests complemented by the Dunn test were applied. All statistical tests were applied with significance level of 5%.

Regarding the sample size, the data of the volumetric analysis of the bone tissue in the proximal face as primary variable, used as reference to confirm the power of the statistical tests, applied in this study. Considering that the minimum difference of averages to obtain statistically significant differences was 10.72%, with a standard deviation of 5.03%, the use of 5 animals of the group allowed the achievement of an α-type error of 0.05 and a β-power of 0.80.

## Results

### Descriptive analysis of right posterior paw joint

**Figure 2:**
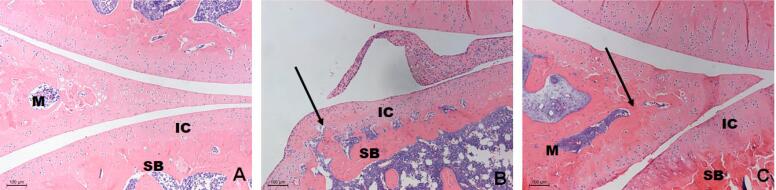
Descriptive analysis of the right posterior paw joints of animals from different groups: A) CTR, B) ART and C) ART/ASU. The arthritis induction promoted alterations in the cartilage articulation, since it was possible to note hyperplasia in the region associated with bone resorption in the subchondral area (black arrow), as observed in Figure B. In Figure C, it can be noticed that the ASU application partially prevented the articular alterations; however, it was still possible to notice changes in the morphology of the articular meniscus (Black Arrow) (Original augmentation 100x-HE). M, meniscus; IC, intra articular cartilage; SB, subchondral bone

There was no loss of animals in this study. The right posterior paw joints of the animals submitted to arthritis induction with alteration of the articular tissues morphology with hyperplasia of the articular cartilage were associated with the resorption of the subchondral bone tissue. ASU administration promoted joint morphology similar to that of the control group, with only minor changes in the articular meniscus morphology (Figure 2).

### Micro CT

**Figure 3:**
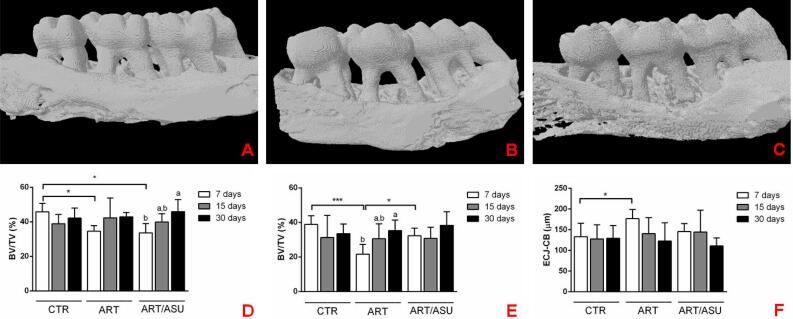
3D-images from micro-CT scans representing all groups at the 7-day period, and the representative graphs of the analyses. A) CTR, B) ART, C) ART/ASU: It is possible to notice the greater degree of bone resorption in the ART group in relation to the other groups. The use of ASU reduced bone resorption in animals with induced arthritis; however, this effect was not enough to reach the same condition of the CTR group. D) BV/TV (%) of the inter-radicular region; E) BV/TV (%) of the interproximal regions; and F) the linear distance from the cementum-enamel junction (CEJ) to the top of the crestal bone (CB) in mm. The CTR group presented higher bone volumes and shorter distance from the CEJ-CB than the ART group. In addition, a greater bone volume was observed in the interproximal regions in the ART/ ASU group in relation to the ART group. All of these differences occurred at the 7-day period. *p<0.05; ***p<0.001 – Significant differences between the groups – One-way ANOVA complemented by Tukey‘s test; Different letters represent different levels of significant differences within each group – One-way ANOVA complemented by Tukey‘s test

Regarding the BV/TV analysis, it was verified that the CTR group presented greater BV/TV at the inter-radicular region (45.83±4.88%) than the other groups at the 7-day period (ART: 34.55±3.20%; ART/ ASU: 33.64±5.33%) (p<0.05), and that the CTR (38.89±5.04 %) and ART/ASU groups (32.24±4.33%) presented higher BV/TV at the interproximal region than the ART group at 7 days (21.61±5.72%) (p<0.05). There was a significant increase in the BV/ TV of the inter-radicular region in the ART/ASU group and of the interproximal region in the ART group at the 30-day period (p<0.05). Compared to the ART group (176.7±22.35 µm), a shorter distance was also shown from the cementum-enamel junction to the top of the bone crest in the CTR group at 7 days (133.3±32.12 µm) (p<0.05) (Figure 3).

### Stereometry

**Figure 4:**
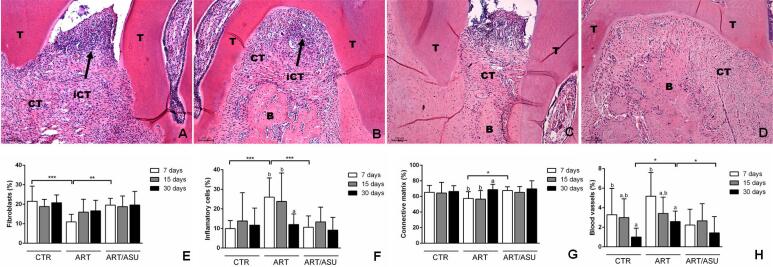
Representative histological images and graphs from the stereometric analysis. A) Interproximal region image representative of the ART group at the period of 7 days, and B) Furcation region image representative of the ART group at the period of 7 days. In both images, the presence of inflammatory infiltrate (black arrows) can be noticed as associated with destruction of bone and reduction of extracellular matrix component and fibroblasts. C) Representative image of the interproximal region of the CTR and ART/ASU groups at the 7-day period; and D) the representative image of the furcation region of the CTR and ART/ASU groups at the 7-day period. The presence of the inflammatory infiltrate may also be noted in these groups, but to a lesser extent than in the ART group. E) Fibroblasts; F) Inflammatory cells; G) Extracellular connective tissue matrix; and H) Blood vessels. In general, the CTR and ART/ASU groups presented more fibroblasts and less inflammatory cells than the ART group at the 7-day period. *p<0.05; **p<0.01; ***p<0.001 – Significant differences between the groups–One-way ANOVA complemented by Tukey‘s test; Different letters represent different levels of significant differences within each group – One-way ANOVA complemented by Tukey‘s test (Original augmentation 100x-HE). B, bone; CT, connective tissue; iCT, inflammatory infiltrate; T, tooth

The CTR and ART/ASU groups presented more fibroblasts (CTR: 21.52±7.79%; ART/ASU: 19.59±3.46%) and less inflammatory cells (CTR: 9.95±4.05%; ART/ASU: 10.66±5.76%) than the ART group (Fibroblasts: 10.95±3.85%; Inflammatory cells: 26.03±9.80%) in the 7-day period (p<0.05). Associated with this event, the ART/ASU group had a higher percentage of extracellular matrix (67.52±4.96%) than the ART group (57.44±8.65%) at 7 days (p<0.05). Compared to the ART group (2.59±1.06%), at 30 days, a smaller number of blood vessels were observed in the CTR (1.00±0.91%), and ART/ASU groups (1.43±1.66 %) (p<0.05) (Figure 4).

### RANKL and TRAP expression

**Figure 5:**
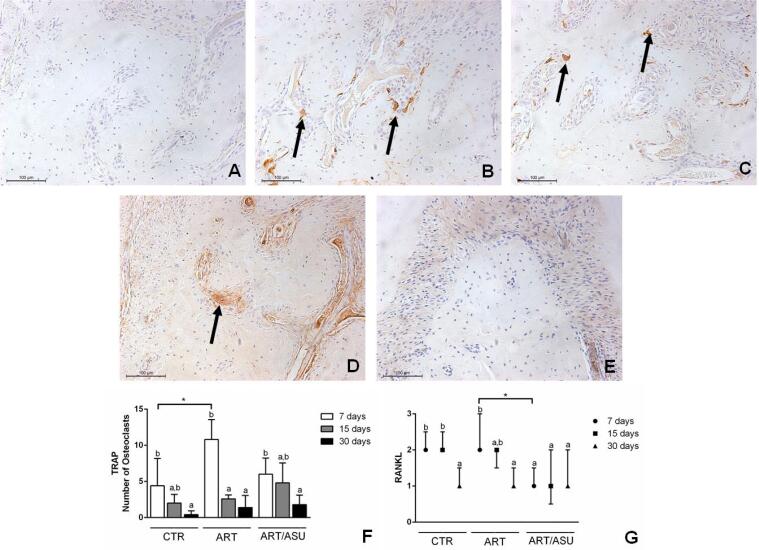
Representative images and graphs of the analysis of TRAP and RANKL expression. A) Negative control; B) TRAP expression in the ART group at 7 days; C) TRAP expression in the CTR group at 7 days. It was shown that the TRAP expression was verified in osteoclast cells that were in the vicinity of the bone tissue (black arrows); D) RANKL expression in the ART group at 7 days; E) RANKL expression at the ART/ASU group at 7 days. The RANKL expression was noted in cells close to the bone tissue or associated with cells in the vicinity of the blood vessels (Black arrows). F) TRAP: Compared with the CTR group at 7 days, a higher expression of TRAP positive cells was shown in the ART group. *p<0.05 – Significant differences between the groups – One-way ANOVA complemented by Tukey‘s test; Different letters represent different levels of significant differences within each group – One-way ANOVA complemented by Tukey‘s test. G) RANKL: Compared with the ART/ASU group, a higher expression of RANKL was observed in the ART group at the period of 7 days. *p<0.05 – Significant differences between the groups – Kruskall-Wallis complemented by Dunn's test; Different letters represent different levels of significant differences within each group – Kruskall-Wallis complemented by Dunn‘s test. (Original augmentation 200x)

At 7 days, the animals in the CTR group presented lower numbers of TRAP positive cells (4.40±3.78%) than the animals of the ART group (10.80±2.77%). The ART/ASU group presented 2-fold lower expression of RANKL than the ART group (p<0.05). In general, the expression of these proteins decreased in all groups with the increase in the evaluation period (Figure 5).

## Discussion

This study generally demonstrated that the induction of arthritis reduced periodontal bone repair in an experimental periodontitis model and that ASU application partially reversed this effect. The BV/TV data and the distance of the cementum/bone crest demonstrated that comparing to the CTR and ART/ASU groups the ART group presented greater loss of bone tissue. Clinical studies have shown that, compared to patients with PD without arthritis, patients with rheumatoid arthritis have more severe PD with worse values in periodontal parameters.^14^^,^^15^^,^^17^ It is important to note that, compared to the ART group, the greater amount of bone volume found in the ART/ASU group corroborates the previous findings showing that ASU promotes an improvement in periodontal repair, although not at the same level as that found in healthy animals in an earlier study.^10^ It is important to note that the mainly statistical differences between the groups occurred at the early periods of evaluation, which may be related with the progressive periodontal repair observed after the ligature removal in this model.^10^

Associated with the results of the bone volume analysis, it was observed that the ART group presented more inflammatory cells and greater expression of TRAP and RANKL than the CTR and ART/ASU groups. The ASU administration has been shown to reduce the expression of pro-inflammatory biological mediators (e.g., IL-1β, TNFα, PGE2, iNOS, and MMPs) in *in vitro* studies with chondroblasts^18^^,^^19^ and fibroblast cells.^20^ These findings have also been demonstrated in preclinical studies in rats, where it was found that ASU administration promoted improved periodontal repair in healthy animals and was associated with reduced expression of important biological mediators of bone resorption, i.e., TRAP, RANKL, and IL-1β.^10^^,^^11^

In addition to the anti-inflammatory effect, ASU has an important effect on connective tissue proliferation and increased expression of growth factors,^21^^–^^23^ and this effect may be related to the results of this study, where there was an improvement in periodontal repair. The proliferative effect of ASU was associated with increased collagen^22^ and aggrecans production.^23^ Associated with this outcome, the ASU application promoted increased expression of growth factors, such as BMP2 in osteoblasts and TGFβ1 in fibroblasts that were previously stimulated with IL-1β.^21^ Preclinical studies have demonstrated that the application of ASU promotes structural changes in the joints of animals that underwent experimental OA.^5^^,^^6^ In clinical studies, it was observed that administration of ASU promotes morbidity reduction and increased mobility of synovial joints, which were associated with reduction of interarticular space in patients with OA.^7^^–^^9^^,^^24^ Oliveira, et al.^12^ (2014) evaluated the effect of ASU on the osseointegration of tibial implants in healthy rats, and their results showed that the higher contact degree between bone-implant in the groups of animals that received the ASU was associated with the increase of the growth factors expression important for the formation of bone tissue (BMP2 and TGFβ1). Additionally, Oliveira, et al.^10^ (2016) demonstrated that ASU administration promotes increased expression of alkaline phosphatase, which is an important marker of osteoblastic activity, and that this event was related with an enhancement in periodontal repair in an experimental periodontitis model in healthy animals.

An important fact is that there were no differences between the CTR and ART/ASU groups in any of the parameters evaluated. This finding corroborates with clinical studies comparing the periodontal condition of patients with and without ART who have a history of periodontal disease, and it does not demonstrate that patients with ART have worse periodontal conditions,^25^^,^^26^ because patients with ART who seek periodontal treatment are usually undergoing some type of therapy with host response modulators, which may at least partially protect the occurrence of significant periodontal bone loss.^26^^–^^28^

This study presents some limitations that must be considered when analyzing the results. The absence of the CTR group with ASU administration does not allow the direct comparison of the ASU effects on healthy animals and with induced arthritis. However, it has been previously shown that, comparing to animals that have received saline solution, the application of ASU in healthy animals promotes an improvement in bone repair^10^ and, therefore, it can be inferred that, compared to animals with induced arthritis also treated with ASU, healthy animals undergoing ASU administration would have better periodontal repair. It will be important to compare the effect of the ASU with different anti-arthritic drugs, with a basically anti-inflammatory function (e.g., bisphosphonates, corticosteroids, anti-TNFα), in induced periodontitis models, focusing on the direct effect on bone repair or resorption, and to evaluate the side effects of each therapy. These studies will be important as they more closely mimic a clinical situation, since patients with rheumatoid arthritis and periodontal disease most often use some sort of anti-arthritic therapy. This comparison may elucidate the real benefits of ASU therapy in patients with arthritis and periodontal disease over other therapies.

Another important discussion is the real impact of ART on the PD pathogenesis. The association of PD and ART has been investigated in cross-sectional studies, and ART has been considered a risk indicator for the presence of PD.^14^^,^^15^ The difficulty of considering ART as a risk factor for periodontal disease is the fact that ART patients are usually under therapy with host response modulators, which modify PD progression.^25^^,^^26^ However, experimental models that induce ART have been shown to increase bone loss in models of experimental periodontitis. In fact, the induced arthritis model used in this study potentiated periodontal bone loss in a model of experimental periodontitis induced by oral inoculation of *Porphyromonas gingivalis* in wild mice and knockout for the IL-17 receptor,^16^ a fact corroborated by this study.

## Conclusion

Considering the results obtained in this study, it can be concluded that, compared to the ART group animals, the use of ASU promoted a significant improvement in periodontal bone repair in animals with experimental arthritis, which was expressed by increased bone volume, reduction in the number of inflammatory cells and reduction of the RANKL and TRAP expression.
